# SRT2104 extends survival of male mice on a standard diet and preserves bone and muscle mass

**DOI:** 10.1111/acel.12220

**Published:** 2014-06-16

**Authors:** Evi M Mercken, Sarah J Mitchell, Alejandro Martin-Montalvo, Robin K Minor, Maria Almeida, Ana P Gomes, Morten Scheibye-Knudsen, Hector H Palacios, Jordan J Licata, Yongqing Zhang, Kevin G Becker, Husam Khraiwesh, José A González-Reyes, José M Villalba, Joseph A Baur, Peter Elliott, Christoph Westphal, George P Vlasuk, James L Ellis, David A Sinclair, Michel Bernier, Rafael de Cabo

**Affiliations:** 1 Translational Gerontology Branch, National Institute on Aging, National Institutes of HealthBaltimore, MD, 21224, USA; 2 Kolling Institute of Medical Research, Royal North Shore HospitalSt Leonards, NSW, 2065, Australia; 3 Sydney Medical School, University of SydneySydney, NSW, 2006, Australia; 4 Division of Endocrinology and Metabolism, Center for Osteoporosis and Metabolic Bone Diseases, University of Arkansas for Medical Sciences and the Central Arkansas Veterans Health Care SystemLittle Rock, AR, 72205, USA; 5 Glenn Labs for the Biological Mechanisms of Aging, Harvard Medical SchoolBoston, MA, 02115, USA; 6 Laboratory of Molecular Gerontology, National Institute on Aging, National Institutes of HealthBaltimore, MD, 21224, USA; 7 Gene Expression and Genomics Unit, National Institute on Aging, National Institutes of HealthBaltimore, MD, 21224, USA; 8 Departamento de Biología Celular, Fisiología e Inmunología, Universidad de Córdoba, Campus de Excelencia Internacional Agroalimentario ceiA3, Campus Rabanales Edificio Severo Ochoa3ª planta, Córdoba, 14014, Spain; 9 Department Physiology, Institute for Diabetes, Obesity, and Metabolism and Perelman School of Medicine, University of PennsylvaniaPA, 19104, USA; 10 Sirtris, a GSK company200 Technology Square, Cambridge, MA, 02139, USA

**Keywords:** healthspan, inflammation, lifespan, muscle wasting, osteoporosis, sirtuins

## Abstract

Increased expression of SIRT1 extends the lifespan of lower organisms and delays the onset of age-related diseases in mammals. Here, we show that SRT2104, a synthetic small molecule activator of SIRT1, extends both mean and maximal lifespan of mice fed a standard diet. This is accompanied by improvements in health, including enhanced motor coordination, performance, bone mineral density, and insulin sensitivity associated with higher mitochondrial content and decreased inflammation. Short-term SRT2104 treatment preserves bone and muscle mass in an experimental model of atrophy. These results demonstrate it is possible to design a small molecule that can slow aging and delay multiple age-related diseases in mammals, supporting the therapeutic potential of SIRT1 activators in humans.

## Introduction

By 2050, there will be 1.5 billion people over the age of 65. This will place a serious burden on global infrastructure and economy. As such, there is an urgent need for treatment modalities to promote healthy aging. The NAD^+^-dependent deacetylase SIRT1 represents an attractive anti-aging target due to its ability to modulate various transcriptional and metabolic pathways (Baur *et al*., 2012). Tissue-specific SIRT1 knock-down in mice leads to pro-inflammatory and metabolic defects (Purushotham *et al*., 2009; Price *et al*., 2012), and whole-body SIRT1 over-expression improves high-fat diet (HFD)-induced metabolic disturbances (Bordone *et al*., 2007; Gillum *et al*., 2011; Li *et al*., 2011) without a beneficial effect on lifespan (Herranz *et al*., 2010). Despite having many cellular targets (Pacholec *et al*., 2010), resveratrol (RSV), a natural polyphenolic SIRT1 activator (Hubbard *et al*., 2013), improves whole-body physiology and lifespan of mice on HFD (Baur *et al*., 2006; Barger *et al*., 2008; Pearson *et al*., 2008) and also has benefits in obese humans (Timmers *et al*., 2011). Synthetic SIRT1 activators with improved selectivity for SIRT1 (Hubbard *et al*., 2013), such as SRT2104, increase insulin sensitivity (Milne *et al*., 2007) and are well tolerated in healthy adults (Hoffmann *et al*., 2013) and elderly volunteers (Libri *et al*., 2012). Small but significant improvements in plasma lipid profiles (Venkatasubramanian *et al*., 2013) and potential for improved insulin sensitivity (Libri *et al*., 2012) have been reported with SRT2104 supplementation. However, these studies are limited by their short treatment time. In this study, the effect of SRT2104 supplementation on health and lifespan in mice on a standard diet was investigated. The health benefits conferred by SRT2104 led us to determine whether short-term treatment could offer protection against disuse atrophy of muscle and bone that occurs in an experimental model of prolonged immobility. Our results suggest that interventions aimed at modulating SIRT1 activity via pharmacological means could represent attractive approaches for delaying the onset of aging and the development of age-related diseases, including sarcopenia and osteoporosis.

## Results and discussion

### SRT2104 treatment improves whole-body physiology and extends lifespan in mice fed a standard diet

**Figure 1:**
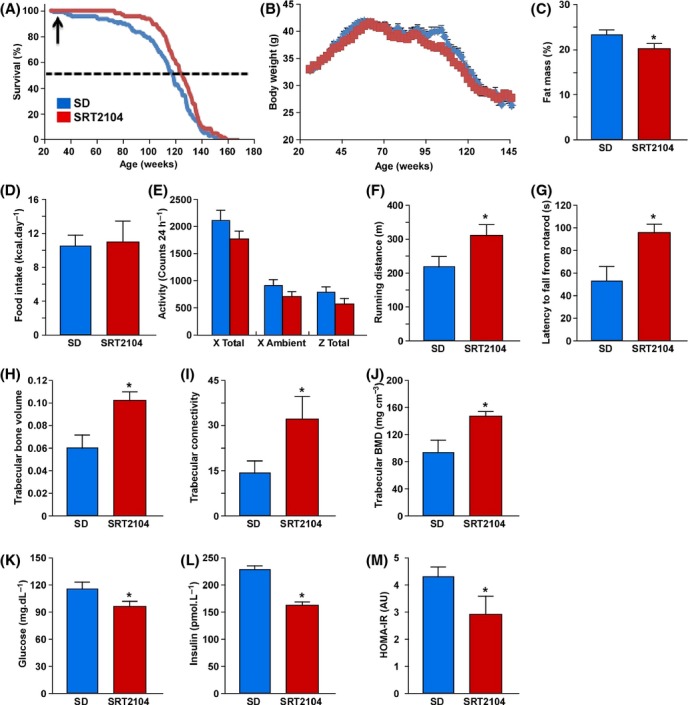
SRT2104 treatment improves whole-body physiology and extends lifespan in mice fed a standard diet. (A) Kaplan–Meier survival curves of mice fed a standard diet (SD) or a SD supplemented with SRT2104. The arrow at 28 weeks indicates the age at which SRT2104 treatment was started. (B–M) The following parameters were analyzed in SD-fed mice without and with SRT2104 supplementation: (B) bodyweights; (C) percentage fat mass; (D) average caloric intake; (E) spontaneous locomotor activity; (F) treadmill performance; (G) time to fall from an accelerating rotarod; (H) trabecular bone volume; (I) trabecular connectivity; (J) trabecular bone mineral density (BMD); (K) circulating glucose and (L) insulin levels were measured after 16 h of fasting; (M) homeostatic measure of insulin resistance (HOMA-IR) index. Data are shown as mean ± SEM. *P < 0.05 compared with SD-fed animals. BV, bone volume; TV, total volume; Tb, trabecular.

To test the effects of the proprietary compound SRT2104, 6-month-old male C57BL/6J mice were placed on a standard AIN-93G diet (SD) supplemented with SRT2104 (100 mg kg^−1^ bodyweight) for the remainder of their lives, which yielded serum concentrations of 261.8 ± 27.0 and 435.7 ± 75.6 ng mL^−1^ in the morning and evening, respectively. SRT2104 supplementation resulted in improved survival of SD-fed mice (χ^2^ = 6.19 and *P* < 0.013) with an increase in mean lifespan of 9.7% (*P* < 0.05) and in maximum lifespan (defined as the 10th percentile) of 4.9% (*P* < 0.001) (Fig. 1A). The immunosuppressant rapamycin has been recently shown to extend maximum lifespan of genetically heterogeneous male mice (Miller *et al*., 2011), and when started at 19 months of age, it also extends lifespan of male and female C57BL/6Nia mice (Zhang *et al*., 2014). Moreover, oral supplementation with the antidiabetic drug metformin leads to healthier and longer life in male mice (Martin-Montalvo *et al*., 2013). The incidences of major pathologies detected at necropsy were reduced with SRT2104 treatment, most notably a trend toward lower prevalence of an enlarged heart and hepatocellular carcinoma with SRT2104, and a significant reduction in peri-renal fat (Table S1, Supporting information). Blinded histological analysis of tissues did not identify any serious pathology in SD mice, and there were no obvious differences between the two groups (Table S2). Consistent with this, biomarkers of liver injury or tissue breakdown were reduced or unchanged after SRT2104 supplementation (Table S3), further confirming that the dose was well tolerated with no obvious toxicity. Interestingly, the increases in longevity induced by SRT2104 occurred despite similar bodyweights between SD-fed controls and treated animals (Fig. 1B). A reduction in percentage fat mass, but not lean body mass, was observed in SRT2104-treated mice (Fig. 1C and Table S4) despite no differences in food consumption (Figs 1D and S1A, Supporting information). Moreover, SRT2104 treatment did not affect spontaneous activity (Fig. 1E), energy expenditure (Fig. S1B), or respiratory exchange ratio (RER) of mice (Fig. S1C). Together, these data indicate that the effects of SRT2104 on lifespan are not due to reduced caloric intake or an increase in voluntary activity. Interestingly, our findings of improved healthspan and modest increases in lifespan are in contrast to whole-body overexpression of SIRT1, which has been shown to result in improvements in healthspan without affecting the longevity of transgenic mice (Herranz *et al*., 2010). It was recently reported that Sirt1 activity in the dorsomedial and lateral hypothalamic nuclei (known as DMH and LH regions) appears to delay aging and promote longevity in both male and female mice (Satoh *et al*., 2013). It is tempting to speculate that some of the beneficial effects of SRT2104 on maximum lifespan stem from its ability to activate SIRT1 present in the DMH and LH hypothalamic regions. Moreover, SRT2104 supplementation was started at 6 months of age, and it is unclear whether treatment at a much younger age would offer similar extension in mouse lifespan. Additional experiments will be required to adequately address these issues. The quality of life of SD-fed mice treated with SRT2104 was then ascertained by monitoring muscle function, balance, and motor coordination. Mice supplemented with SRT2104 exhibited significant improvement in endurance performance on the treadmill (Fig. 1F) and better motor skills, as assessed by rotarod performance (Fig. 1G).

Bone health was also assessed as osteoporosis leads to increased rates of morbidity and mortality in the elderly due to a decrease in bone strength and increased risk of fractures (Gass & Dawson-Hughes, 2006; Lyles *et al*., 2007). In the distal femur of adult mice, SRT2104 significantly improved trabecular bone volume, trabecular connectivity, and trabecular bone mineral density compared with control SD-fed animals (Fig. 1H–J); however, no effect in cortical thickness was observed (data not shown). Overall, SRT2104 improved a number of parameters involved in bone health and suggests that SRT2104 may be a countermeasure for age-related bone loss.

One hallmark of the aging process is the impairment of glucose homeostasis that leads to type 2 diabetes and cardiovascular diseases. Therefore, we next explored the effect of SRT2104 on whole-body metabolism in mice on SD. Fasting blood glucose and insulin levels, and insulin resistance index, as determined by homeostasis model assessment of insulin resistance (HOMA-IR), were all significantly reduced in SRT2104-treated mice (Fig. 1K–M). Apparent improvements in the oral glucose tolerance test (OGTT) and insulin tolerance test (ITT) with SRT2104 did not reach statistical significance (Fig. S1D,E). It is likely that clamp studies would have provided solid evidence of the beneficial metabolic effects of SRT2104. SRT2104 supplementation was accompanied by a trend toward reduced serum free-fatty acid (FFA) levels with no change in circulating triglycerides or total cholesterol levels (Table S3). SIRT1 overexpression increases fatty acid beta-oxidation (Purushotham *et al*., 2009), and it is likely that SRT2104 supplementation will also have an impact on this pathway. While the increases in beta-oxidation reported by Purushotham *et al*. (2009) stemmed from lentiviral infection of primary hepatocytes from liver-specific SIRT1-KO mice with recombinant SIRT1 (Fig. 1D), this experimental model was markedly different than our use of wild-type mice fed a standard diet. Therefore, the changes observed by Purushotham *et al*. (2009) may not be directly translatable to our study. It is interesting to note that short-term SRT2104 supplementation was associated with improved lipid profile in healthy cigarette smokers (Venkatasubramanian *et al*., 2013), which is consistent with our findings. Overall, our results show for the first time that SRT2104 prolongs lifespan, improves whole-body metabolic function, and delays the onset of age-related diseases in SD-fed male mice.

### SRT2104 treatment increases mitochondrial content and suppresses the inflammatory response

**Figure 2:**
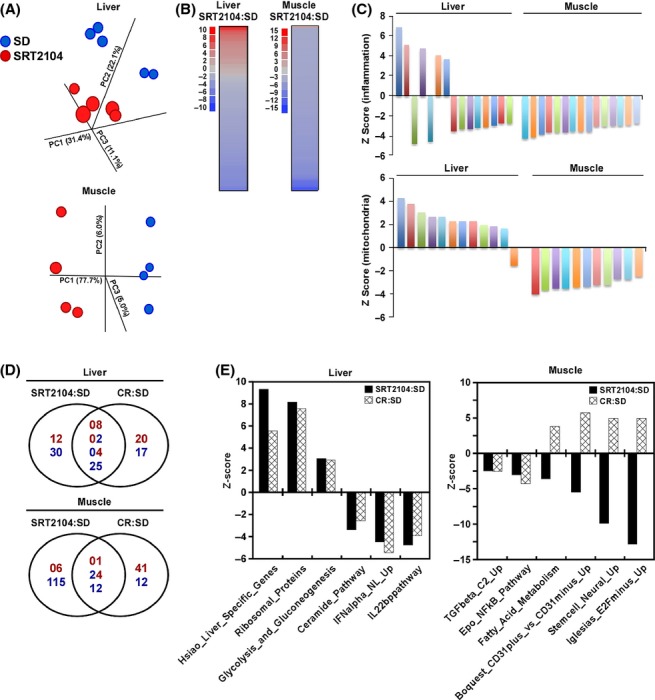
SRT2104 changes the gene expression profile differently in liver and muscle. (A) Principal component analysis (PCA) was performed on liver and muscles tissues of mice fed a SD or SD supplemented with SRT2104. (B) Parametric analysis of gene-set enrichment (PAGE) analysis was performed on microarray data from mice fed a SD or subjected to SRT2104. Columns show pathways significantly upregulated (red) or downregulated (blue) by SRT2104 treatment. See also Tables S5–S7. (C) Effect of SRT2104 on inflammatory and mitochondrial-related pathways from the PAGE analysis for liver and skeletal muscle. (D) Venn diagrams of overlapping gene sets significantly modified by SRT2104 vs. calorie restriction (CR). Upregulated gene sets are depicted in red and the downregulated gene sets in blue. (E) Effect of SRT2104 and CR on select gene sets from mouse liver and muscle. The list of the significantly modified gene sets can be found in Tables S6 and S7. SD, standard diet.

To further gauge the molecular mechanisms by which SRT2104 improved whole-body metabolism and survival in SD-fed mice, a whole-genome microarray analysis was performed on liver and muscle tissues. Principal component analysis (PCA) showed a distinct separation of both treatment groups, with the effect more pronounced in the muscle tissue (Fig. 2A). For both the liver and muscle, the largest changes induced by SRT2104 treatment are presented in Table S5, and the complete dataset is available at http://www.ncbi.nlm.nih.gov/geo/. Notably, transcripts belonging to the cytokine-induced STAT inhibitor (CIS) family were upregulated in the liver [suppressor of cytokine signaling 2 (SOCS2)] and muscle [cytokine-inducible SH2-containing protein (CISH)] of SRT2104-treated mice, consistent with suppression of the inflammatory response. Moreover, within the highest affected transcripts, an upregulation of albumin D-box binding protein (DBP) gene was found across both tissues. Interestingly, DBP is a clock-controlled gene, whose circadian expression is regulated by SIRT1 (Nakahata *et al*., 2008). To further investigate the effect of SRT2104 in modifying liver and skeletal muscle gene expression, we next performed parametric analysis of gene-set enrichment (PAGE). Pathways that were altered by SRT2104 supplementation are graphically represented in Fig. 2B and indicated that the transcriptional effect of SRT2104 was stronger in muscle than in liver. Downregulation occurred for the large majority of the modified pathways in response to SRT2104, which included gene sets such as ‘inflammation’ and ‘mitochondrial metabolism’ (Fig. 2C). The interpretation of microarray data has benefited from comparison with the effects of calorie restriction (CR). Among the top twenty genes whose expression was modified by SRT2104, more than 70% (14/20) were responsive to CR in the liver but less than 40% (8/20) in skeletal muscle (Table S5). The gene encoding Txnip, a negative regulator of mTORC1-mediated protein translation (Jin *et al*., 2011), was upregulated in the liver of SRT2104- and CR-treated mice, whereas the hepatic expression of *Elovl3*, encoding a condensing enzyme that provides precursors for ceramide synthesis (Park *et al*., 2010), was significantly lower in response to Sirt1 induction by SRT2104 or CR. Moreover, *Cish* expression in the mouse muscle was upregulated by CR, with a Z-ratio of 11.58 (Table S5). There were 81 and 76 gene sets that were significantly modified by SRT2104 and CR, respectively, in mouse liver when compared to SD-fed animals. Of these, 39 gene sets were shared with the majority (25/39) being downregulated by both interventions (Fig. 2D). Mouse skeletal muscle had more than 158 and 90 gene sets that were significantly affected by SRT2104 and CR, respectively, of which 37 gene sets were shared. Interestingly, ~32% (12/37) of these pathways were downregulated by both interventions, while ~65% (24/37) were reciprocally altered by CR and SRT2104 (Fig. 2D). The complete list of overlapping gene sets is presented in Table S6 (liver) and Table S7 (muscle). Among the gene sets that were modified in the same direction in liver included ‘Ribosomal_proteins’ and ‘Ceramide_Pathway’, whereas reciprocal regulation of gene sets by CR and SRT2104 in muscle included ‘Boquest_CD31plus_vs_CD31minus_Up’, ‘Stemcell_Neural_Up’, and ‘Iglesias_E2Fminus_Up’ (Fig 2E). Resident muscle stem cell side population as defined as CD31 (Pecam-1) negative lineage (Motohashi *et al*., 2008) might be reciprocally affected by SRT2104 or CR (Schmuck *et al*., 2011).

The effect of SRT2104 resulted in an overall downregulation of inflammatory pathways in the muscle, while being more complex in the liver. Nevertheless, the expression profile of several genes controlled by the pro-inflammatory NF-κB transcription factor exhibited a pattern that was largely comparable between SRT2104 and CR treatment in liver (Table S8) and muscle (Table S9). As anticipated, SRT2104 supplementation significantly lowered serum TNF-α and MCP-1 levels as compared to controls (6.1 ± 0.7 vs. 3.9 ± 0.5 and 72.0 ± 8.9 vs. 47.9 ± 8.9 pg mL^−1^, respectively; *P* < 0.05).

**Figure 3:**
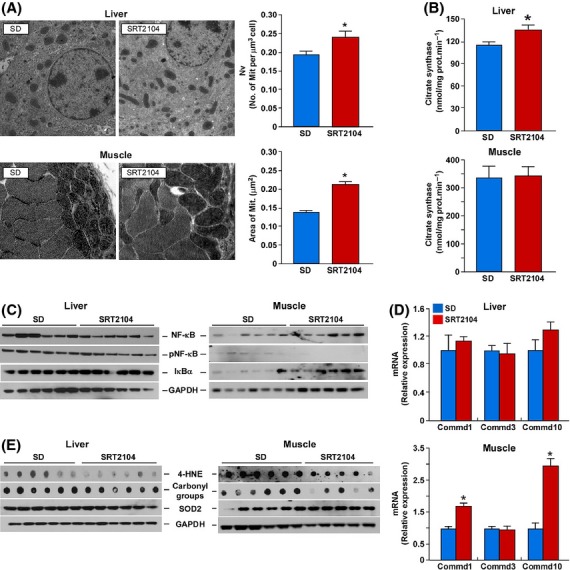
SRT2104 treatment increases mitochondrial content and suppresses the inflammatory response. (A) Representative transmission electron micrographs of liver and muscle, and the respective mitochondrial quantification. (B) Citrate synthase activity. (C) Representative immunoblots from inflammatory markers in liver and muscle tissues. (D) mRNA levels of COMMD genes assessed by quantitative real-time PCR. Relative expression values were normalized to SD-fed mice. (E) Representative immunoblots from oxidative stress markers in liver and muscles. Data are shown as mean ± SEM. *P < 0.05 compared with SD-fed mice.

Intriguingly, a reciprocal pattern of expression of genes related to mitochondrial metabolism was observed between the liver and muscle, indicating that the effects of SRT2104 were tissue-specific. In agreement with the microarray data, transmission electron microscopy revealed higher mitochondrial content in the liver of SRT2104-fed mice (Fig. 3A), which correlated with increased citrate synthase activity (Fig. 3B). In contrast, mitochondrial size was significantly higher in muscle of SRT2104-fed mice despite no change in citrate synthase activity (Fig. 3A,B). In both liver and muscle, expression of subunits of ETC protein complexes was either unaltered or slightly downregulated after SRT2104 treatment (Fig. S2).

The anti-inflammatory effects of SRT2104 that were observed in SD-fed mice correlated with defect in NF-κB-induced gene expression (Table S8). The ratio of phospho-active to total form of RelA/p65 fell in the muscle of SRT2104-treated mice, while being unaffected in the liver of these animals (Fig. 3C). This reduction in ‘active’ NF-κB coincided with significant increase in IκBα levels (Fig. 3C). A critical mechanism of transcriptional suppression of NF-κB involves the copper metabolism MURR1 domain containing (COMMD) proteins through promotion of the ubiquitination and degradation of NF-κB subunits (Maine *et al*., 2007). Analysis of COMMD gene expression revealed a significant increase in the mRNA levels of COMMD1 and COMMD10 in muscle, but not in liver, of SRT2104-treated mice compared with control SD-fed mice (Fig. 3D). RelA/p65 protein levels were upregulated in C2C12 myoblasts in response to SRT2104 treatment (Fig. S3), in agreement with the increased expression of RelA/p65 in muscle of SRT2104-treated mice (Fig. 3C). The transactivation potential of NF-κB is modulated by phosphorylation and acetylation (Hayden & Ghosh, 2008) whereby Sirt1-mediated deacetylation of RelA/p65 causes a decrease in NF-κB transcriptional activity (Yeung *et al*., 2004). Here, SRT2104 led to lower acetylation of RelA/p65 in C2C12 myoblasts (Fig. S3), likely due to selective activation of SIRT1 (Hubbard *et al*., 2013). These findings together with the microarray data suggest that SRT2104 suppresses NF-κB activity partly through increase in COMMD expression and reduction in RelA/p65 acetylation.

Oxidative stress activates NF-κB, while SIRT1 activation increases the antioxidant response (Salminen *et al*., 2013). Here, protein carbonylation and formation of 4-HNE adduct, a marker of lipid peroxidation, were significantly reduced in the liver and muscle of SRT2104-treated mice, with the effect being more pronounced in muscle (Fig. 3E). The levels of the antioxidant protein superoxide dismutase (SOD2) were unchanged in the liver, but increased in muscles of SRT2104-treated animals (Fig. 3E). The antioxidant capacity of SRT2104 may represent a compensatory stress signal triggered in response to age-dependent, ROS-mediated mitochondrial dysfunction in mice, with a predominant effect of SRT2104 on mitochondria of skeletal muscle. This adaptive response called mitochondrial hormesis has been found to promote longevity in Drosophila (Owusu-Ansah *et al*., 2013).

### Short-term SRT2104 treatment preserves muscle and bone mass

**Figure 4:**
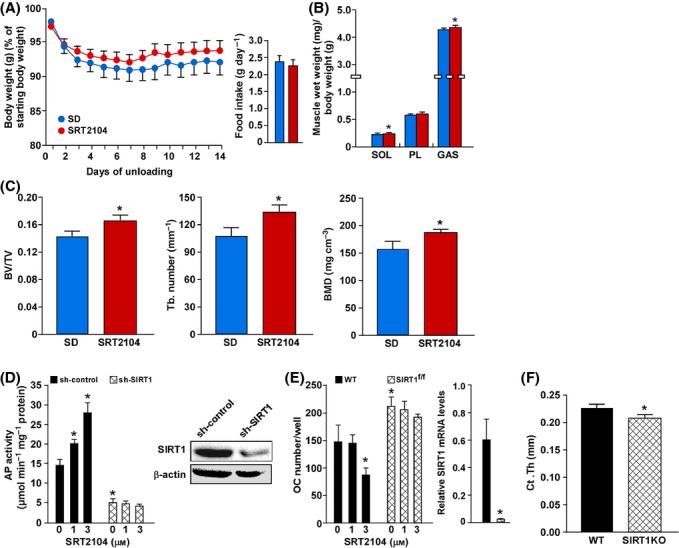
Short-term SRT2104 treatment preserves muscle and bone mass. (A) Bodyweights during 14 days of hindlimb suspension and average food consumption (inset) for 6-month-old mice fed either a standard diet (SD) or SD supplemented with SRT2104 for 6 weeks. (B) Muscle weights. (C) Trabecular bone volume, trabecular connectivity, and trabecular bone mineral density (BMD). (D) Alkaline phosphatase (AP) activity in C2C12 cells infected with SIRT1 shRNA or nontargeting shRNA control and treated with 1 and 3 μm SRT2104 for 24 h. (E) Osteoclast (OC) number in bone marrow-derived osteoblastic cells from wild-type (WT) mice and SIRT1^f/f^ mice and treated with 1 and 3 μm SRT2104 for 4 days. (F) Cortical thickness in femurs from wild-type (WT) and SIRT1KO mice. Data are mean ± SEM. *P < 0.05. SOL, soleus; PL, plantaris; GAS, gastrocnemius; BV, bone volume; TV, total volume; Tb, trabecular.

Short-term effects of SRT2104 were examined using the hindlimb suspension, a well-established model of muscle atrophy (Sandri *et al*., 2004). SRT2104 supplementation did not affect bodyweights or food consumption during 2 weeks of unloading (Fig. 4A). However, the induced loss of muscle mass for both the soleus and gastrocnemius muscles was attenuated in the SRT2104 cohort (Fig. 4B). Using a second model of atrophy, where endocrine signals rather than inactivity promote muscle loss, both the soleus and tibialis muscles from SRT2104-treated mice were found to be more resistant to fasting-induced atrophy (Fig. S4A). The involvement of NF-κB in the control of muscle size and strength (Cai *et al*., 2004; Mourkioti *et al*., 2006) and the ability of SIRT1 activators to attenuate NF-κB signaling led us to examine expression of pro-inflammatory mediators and RelA/p65 levels following short-term supplementation with SRT2104. Using the hindlimb-unloading model, SRT2104 treatment did not affect RelA/p65 protein levels, but led to an increase in PGC-1α levels (Fig. S4B). The latter observation may partly explain the protection against muscle atrophy through suppression of FOXO3-mediated induction of the atrogenes MuRF-1 and atrogin-1 (Sandri *et al*., 2006). In addition, AKT is known to suppress FOXO3 transcriptional activity (Milne *et al*., 2007), although data presented here do not show alterations in pAKT protein levels after SRT2104 treatment (Fig. S4B). To further elucidate the role of SIRT1 in muscle atrophy, young transgenic mice with muscle-specific SIRT1 knock-down (mSIRT1KO) were subjected to 2 weeks of hindlimb suspension. When compared with wild-type animals, suspended mSIRT1KO mice exhibited a significant increase in skeletal muscle atrophy (Fig. S4C). These results indicate that SIRT1 activation via short-term supplementation with SRT2104 alleviates muscle loss in hindlimb-unloading model in mice.

Unloading is also known to cause disuse osteoporosis (Sandri *et al*., 2004). SRT2104-treated mice subjected to hindlimb suspension had higher trabecular bone volume, trabecular connectivity, and trabecular bone mineral density, but not cortical bone mass (data not shown), compared with the SD-fed control mice (Fig. S4C). To better evaluate the specificity of SRT2104 action, C2C12 myoblasts were stably transfected with small hairpin RNA to knock-down SIRT1 and then examined for alkaline phosphatase (AP) activity, a marker for osteogenic differentiation. The ability of SRT2104 to increase AP activity was totally dependent on SIRT1 expression (Fig. 4D). Similarly, the proliferation rate was markedly reduced after SRT2104 treatment of C2C12 myoblasts (Fig. S4D). In a second series of experiments, mineralization in bone marrow-derived osteoblastic cells was increased, while the number of osteoclasts was decreased upon treatment of wild-type mice with SRT2104 (Fig. S4E and Fig. 4E). This action of SRT2104 was not observed in cells derived from mice lacking SIRT1 (SIRT1^f/f^) (Fig. 4E). The role of SIRT1 in bone remodeling was further confirmed in whole-body SIRT1KO mice showing reduced cortical bone thickness compared with wild-type mice (Fig. 4F). This is in agreement with previous reports showing the beneficial role of SIRT1 in regulating bone mass (Cohen-Kfir *et al*., 2011; Edwards *et al*., 2013). Thus, SRT2104 affects both features of age-related osteoporosis by increasing bone formation and suppressing bone resorption in a SIRT1-dependent manner.

We acknowledge the limitations of using male mice only, and the fact that some experiments involved small numbers of animals. Nevertheless, we provide novel evidence for the beneficial effects of SIRT1 activators on healthspan and lifespan in male mice maintained on standard diet. Moreover, SRT2104 may have therapeutic utility against sarcopenia and involutional and disuse-mediated osteoporosis.

## Experimental procedures

### Longevity study animals

Male C57BL/6J mice were obtained from the Jackson Laboratory (Bar Harbor, ME, USA) and housed at the Gerontology Research Center (Baltimore, MD, USA). Mice were housed in cages of four with *ad libitum* access to diet and tap water. Mice were electronically tagged for identification (Biomedic Data System Inc., Maywood, NJ, USA), and bodyweight and food intake were monitored twice monthly. Mice were not fasted prior to sacrifice. Animal rooms were maintained at 20–22 **°**C with 30–70% relative humidity and a 12-hour light/dark cycle. All animal protocols were approved by the Animal Care and Use Committee (325-LEG-2012) of the National Institute on Aging.

### Generation of a whole-body SIRT1 knockout mouse

Mice harboring a Cre-ERT2 fusion protein were crossed to SIRT1^**Δ**ex4^ mice (Cheng *et al*., 2003) to generate SIRT1^**Δ**ex4^ERT2 mice in which the catalytic region of SIRT1 can be deleted upon treatment with tamoxifen, as described previously (Price *et al*., 2012). Cre induction was carried out by i.p. injection of tamoxifen citrate (1 mg mouse^−1^ per day) for 5 days. Mice were not fasted prior to sacrifice. Western blots have been performed to confirm successful reduction in SIRT1 protein expression in whole-body SIRT1-KO mice (Price *et al*., 2012).

### Generation of a muscle-specific adult-inducible SIRT1 knockout mouse

An adult-inducible muscle-specific SIRT1 knockout mouse was generated by crossing mice with a Cre recombinase transgene under the control of the human skeletal actin promoter (HSA-Cre) with SIRT1^**Δ**ex4^ mice. Cre induction was carried out by i.p. injection with tamoxifen citrate (1 mg mouse^−1^ per day) for 5 days. Mice were not fasted prior to sacrifice. Deletion of SIRT1 was confirmed by Western blotting (data not shown).

### Hindlimb suspension study

At 5 months of age, mice were housed individually and suspended by the tail using a strip of adhesive surgical tape attached to a nylon monofilament line via a stainless steel swivel. Mice were suspended at a 30° angle to the floor with only the forelimbs touching the floor. The swivel enabled the animal to explore the cage (360° range of motion) and obtain food and water freely. Food consumption and bodyweight were recorded daily, and the angle of suspension was adjusted if necessary. Following 14 days of suspension, mice were euthanized and soleus, plantaris, and gastrocnemius muscles were collected using standardized dissection methods. Mice were not fasted prior to sacrifice. Muscle tissue was cleaned of excess fat and connective tissue, weighed on an analytical balance, and collected for further analysis (*n* = 10 SD, *n* = 10 SRT2104, 26 weeks age, 6 weeks diet; muscle-specific SIRT1 knockout (mSIRT1 KO) mice: *n* = 10 wild-type, *n* = 10 mSIRT1 KO, 22 weeks age).

### 48-h fasting study

Male C57BL/6 mice were obtained from the Jackson Laboratory (Bar Harbor) at 4 months of age. They were fed house chow (Harlan Teklad Global 18% Protein Rodent Diet; Harlan Teklad, Madison, WI, USA) until they reached 7 months of age at which time they were fed either a standard AIN-93G diet (SD; carbohydrate:protein:fat ratio of 64:19:17 percent of kcal) or SD supplemented with SRT2104 (100 mg kg^−1^) for 6 weeks. Then, mice were moved to clean cages and had food removed for 48 h. Water was available *ad libitum* throughout this time. Following 48 h of fasting, mice were euthanized and soleus, plantaris, gastrocnemius, tibialis, and extensor digitorum longus were dissected using standardized dissection methods. Individual muscles were weighed and frozen for further analysis (*n* = 7 SD, *n* = 7 SRT2104; 40 weeks age, 12 weeks diet).

### Diets

For the longevity study, diets were started at 28 weeks of age after randomization into two groups of 100 mice per group. Mice were fed a standard AIN-93G diet (SD; carbohydrate:protein:fat ratio of 64:19:17 percent of kcal), or a SD supplemented with SRT2104. SRT2104 was added at a dose of 1.33 g drug per kg of chow, formulated to provide daily doses of ~ 100 mg drug kg^−1^ bodyweight. The longevity study diets were purchased from Dyets, Inc. (Bethlehem, PA, USA), and SRT2104, a proprietary compound, was provided by Sirtris Pharmaceuticals, Inc. (Cambridge, MA, USA). For the hindlimb suspension study, starting at 4 months of age, C57BL/6 mice were fed either a standard AIN-93G diet (SD; carbohydrate:protein:fat ratio of 64:19:17 percent of kcal) or a SD supplemented with SRT2104 for 4 weeks prior to suspension, and then for an additional 2 weeks during the suspension. Diets were formulated so mice received a daily dose of 200 mg drug kg^−1^ of bodyweight. Diets were supplied directly to us by Sirtris Pharmaceuticals, Inc. For the 48-h fasting study**,** mice were fed either SD or a SD supplemented with SRT2104 (100 mg kg^−1^) for 6 weeks prior to sacrifice. This is the same diet as for the longevity study mice. For the whole-body SIRT1 knockout and muscle-specific SIRT1 knockout mouse models, mice were maintained on house chow (Teklad Global 18% Protein Rodent Diet; Harlan Teklad; carbohydrate:protein:fat ratio of 58:24:18 percent of kcal) for the course of their lives.

### Survival study

Animals were inspected daily for health issues, and deaths were recorded for each animal. Moribund animals were euthanized, and every animal found dead or euthanized was necropsied. Criteria for euthanasia were based on an independent assessment by a veterinarian according to the AAALAC guidelines. For the longevity study, only cases where the condition of the animal was considered incompatible with continued survival are represented as deaths in the curves. Animals removed at sacrifice or euthanized due to reasons not related to incompatible survival were considered as censored deaths. In the standard diet group, 18 mice were censored due to dermatitis (*n* = 3; 80 weeks age, 97 weeks age, 112 weeks age), paralysis (*n* = 1; 37 weeks age), growth per mass (*n* = 1; 105 weeks age), or experimental procedures (*n* = 13; 40 weeks age, 115 weeks age), leaving 83 mice for the survival study. Of the 18 mice censored, only five were euthanized. In the SRT2104 group, 14 mice were censored due to vets orders (*n* = 2; 74 weeks age, 97 weeks age), prolapsed anus (*n* = 1; 79 weeks age), or experimental procedures (*n* = 11; 40 weeks age, 115 weeks age), leaving 86 mice for the survival analysis. Of the 14 mice censored, three were euthanized.

### Body composition

Measurements of lean, fat, and fluid mass in live mice were acquired by nuclear magnetic resonance (NMR) using the Minispec LF90 (Bruker Optics, Billerica, MA, USA) (*n* = 15 SD, *n* = 13 SRT2104; 76 weeks age; 49 weeks diet).

### Metabolic assessment

Mouse metabolic rate was assessed by indirect calorimetry in open-circuit oxymax chambers using the Comprehensive Lab Animal Monitoring System (CLAMS; Columbus Instruments, Columbus, OH, USA) as described previously (Minor *et al*., 2011) (*n* = 8 SD, *n* = 8 SRT2104; 56 weeks age, 29 weeks diet).

### Physical performance

All mice were acclimated to the testing room for 15 min prior to the commencement of any testing. Rotarod and treadmill methodologies are provided in the supplemental materials.

### Oral glucose tolerance test (OGTT)

Following an overnight fast, mice received a 30% glucose solution (2 g kg^−1^ glucose by gavage). Blood glucose was measured using an Ascensia Elite glucose meter (Bayer, Mishawaka, IN, USA) at 0, 15, 30, 60, and 120 min following gavage (*n* = 6 SD, *n* = 8 SRT2104; 73 weeks age; 46 weeks diet).

### Insulin tolerance test (ITT)

Following a 3-h fast, mice received an i.p. injection of human insulin (1.5 IU kg^−1^; Novo Nordisk Inc., Plainsboro, NJ, USA). Blood glucose was measured using an Ascensia Elite glucose meter (Bayer) at 0, 15, 30, 60 and 120 min (*n* = 4 SD, *n* = 7 SRT2104; 70 weeks age; 30 weeks diet).

### Serum markers and HOMA calculation

Information can be found in the supplemental section.

### Histology

Mice were euthanized and organs fixed for histological analysis in 4% paraformaldehyde. Tissues were embedded in paraffin and stained with hematoxylin and eosin. Pathology was scored by a qualified pathologist blinded to diet and treatment group (*n* = 6 SD, *n* = 6 SRT2104; 81 weeks age, 41 weeks diet).

### Electron microscopy

Liver and skeletal muscle (gastrocnemius) from mice were removed and placed directly into a fixative solution consisting of 2.5% glutaraldehyde and 3% paraformaldehyde in 0.1 m sodium cacodylate buffer (Electron Microscopy Sciences, Hatfield, PA, USA). Additional information can be found in the supplemental materials (*n* = 3 SD, *n* = 3 SRT2104; 81 weeks age, 41 weeks diet).

### Microarray

Principal components were calculated using diane 6.0 software (http://www.grc.nia.nih.gov/branches/rrb/dna/diane_software.pdf). For the calculation of pairwise distances between samples, each microarray was considered as a point in a high-dimensional space because we treated each probe as a variable. Parametric analysis of gene-set enrichment (PAGE) was analyzed as previously described (Kim & Volsky, 2005). All raw data are available in the Gene Expression Omnibus database (Accession No. GSE49000) (*n* = 4–5 per group; 41 weeks age, 14 weeks diet).

### PCR

Detailed information can be found in the supplemental section.

### Western blotting

Detailed information can be found in the supplemental section.

### Citrate synthase activity

Citrate synthase activity was determined in ~20 μg of protein lysates following the method described by Bernier *et al*. (2011). Citrate synthase were determined using spectrophotometric methods. Results were expressed in nmol mg^−1^ protein per min (*n* = 6 SD, *n* = 6 SRT2104; 81 weeks age, 41 weeks diet).

### C2C12 cell culture conditions and treatment

C2C12 cell line (ATCC, Manassas, VA, USA) was cultured in low glucose Dulbecco’s modified Eagle’s medium (DMEM) (Invitrogen, Carlsbad, CA, USA) supplemented with 10% fetal bovine serum (FBS; Invitrogen) and penicillin–streptomycin (Invitrogen). Cells were treated with vehicle (0.1% DMSO) or 3 μm SRT2104 for 24 h and then harvested for protein and Western blotting using methods detailed elsewhere.

### Bone imaging

Femurs were loaded into 10 mm diameter scanning tubes and imaged with a Scanco microCT40 instrument (CT40, Scanco Biomedical, Bruttisellen, Switzerland) as previously described (Jilka *et al*., 2010; Martin-Millan *et al*., 2010). Cortical and trabecular bone measurements were analyzed as previously described (Martin-Millan *et al*., 2010; Onal *et al*., 2012). Additional information can be found in the supplemental section.

### Osteoclast formation and adenovirus infection

Macrophages were developed from bone marrow cells flushed from the femurs of three wild-type or *SIRT1*^*f/f*^ *mice* (Jackson Laboratories, Bar Harbor, ME, USA) cultured in α-MEM supplemented with 10% FBS and 1% PSG (Invitrogen) in the presence of 30 ng mL^−1^ M-CSF (R&D Systems, Minneapolis, MN, USA). Four days later, cells were infected with adenovirus encoding Cre recombinase (Ad-Cre) (Vector Biolabs, Philadelphia, PA, USA) at a MOI of 30 for 6 h. Seventy-two hours later, cells were trypsinized and replated in 48-well plates and cultured for 4 days with 30 ng mL^−1^ M-CSF and 30 ng mL^−1^ RANKL (R&D Systems) to obtain osteoclasts, in the presence of vehicle or SRT2104. At the end of the experiment, osteoclasts were fixed with 10% neutral-buffered formalin for 15 min and stained for tartrate-resistant acid phosphatase (TRAP). Multinuclear TRAP+ cells were quantified.

### Alkaline phosphatase (AP) activity and SIRT1 silencing

C2C12 cells were cultured in DMEM supplemented with 10% FBS, 1% each penicillin, streptomycin, and glutamine, and 1% sodium pyruvate. Expression of SIRT1 was knocked down by transduction with lentiviruses encoding shRNA to Sirt1 (NM_019812) according to the manufacturer’s protocol (Sigma-Aldrich, St. Louis, MO, USA). C2C12 cells transduced with a nontarget shRNA (SHC002V) were used as control. After selection with puromycin (2500 ng mL^−1^) for 14 days, the SIRT1-silenced cells were seeded at 2 × 10^4^ cm^−2^ in medium containing 10% FBS. The following day, the medium was replaced with 5% serum-containing medium. Cells were lysed in 100 mm glycine, 1 mm MgCl_2,_ and 1% Triton X-100 at pH 10. AP activity in cell lysates was determined using a buffer containing 2-amino-2-methylpropanol and *p*-nitrophenylphosphate (Sigma-Aldrich). Alkaline phosphatase activity was normalized to protein content, which was determined using a Bio-Rad DC protein assay kit (Hercules, CA, USA).

### Mineralization assay

Detailed information can be found in the supplemental section.

### Proliferation assay

Detailed information can be found in the supplemental section.

### Statistics

Data are expressed as means ± standard error of the mean (SEM). Student’s t-tests were used for all comparisons. Mortality during the survival study was assessed through the use of the log-rank test to compare the differences in Kaplan–Meier survival curves. Maximal lifespan was defined as the 10th percentile of mice still alive. Analyses were performed using Excel 2010 (Microsoft Corp., Redmond, WA, USA), IBM SPSS Statistics (Amonk, NY, USA), or sigmastat 3.0 (Aspire Software International, Ashburn, VA, USA). A *P* value of ≤ 0.05 was considered statistically significant.

## Supporting Information

**Fig. S1** Effects of SRT2104 supplementation on various metabolic parameters in mice on a standard diet (related to Fig. 1).

**Fig. S2** Representative immunoblots of mitochondrial complexes in liver and muscle from mice on a standard diet (SD) and SD supplemented with SRT2104 (related to Fig. 3).

**Fig. S3** SRT2104 reduces p65/RelA acetylation levels in C2C12 cells.

**Fig. S4** Impact of SRT2104 supplementation on muscle and bone health (related to Fig. 4).

**Data S1** Material and methods. **Table S1** Major gross pathologies identified at necropsy. **Table S2** Blinded histopathological analysis. **Table S3** Effect of SRT2104 on various biomarkers in serum. **Table S4** Body composition. **Table S5** The ten most highly upregulated and downregulated genes, based on Z-ratio in liver and muscles of SRT2104-treated mice compared with standard diet (SD). **Table S6** List of pathways significantly modified by SRT2104 and CR in the liver of SD-fed mice. **Table S7** List of pathways significantly modified by SRT2104 and CR in muscle of SD-fed mice. **Table S8** Significant expression of a set of NF-κB target genes in the liver of SRT2104- vs. CR-treated mice. **Table S9** Significant expression of a set of NF-κB target genes in skeletal muscle of SRT2104- vs. CR-treated mice. **Table S10** List of primer sequences used for quantitative PCR analysis.
