# Supplementary figures and images for: SRT2104 extends survival of male mice on a standard diet and preserves bone and muscle mass
Source: Aging Cell. 2014 Jun 16;13(5):787–96. doi: 10.1111/acel.12220 (PMC4172519; doi:10.1111/acel.12220)

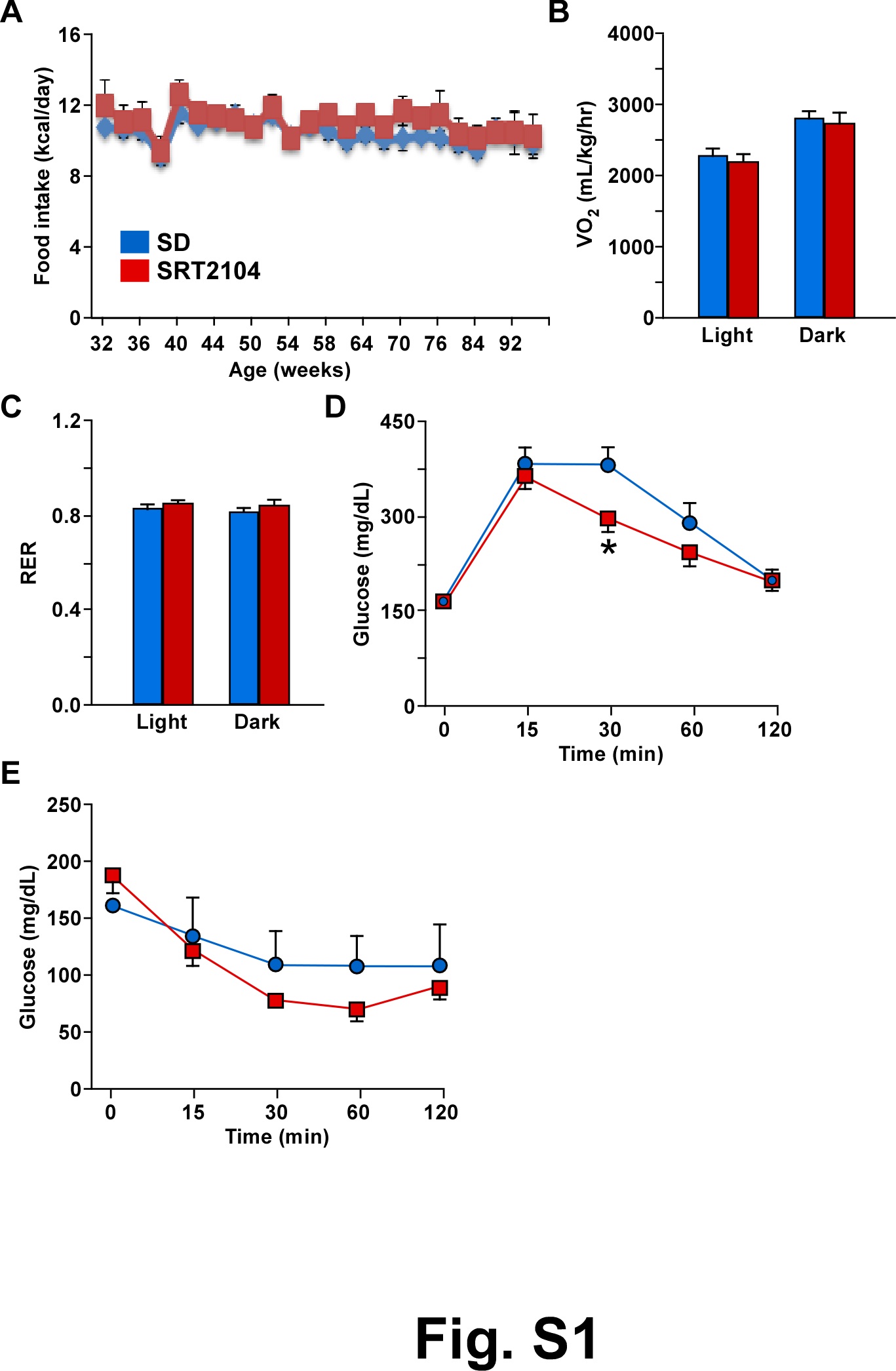

Supplement: Supplementary file 1 — Fig. S1 Effects of SRT2104 supplementation on various metabolic parameters in mice on a standard diet (related to Fig. 1). [file acel0013-0787-sd1.jpg]

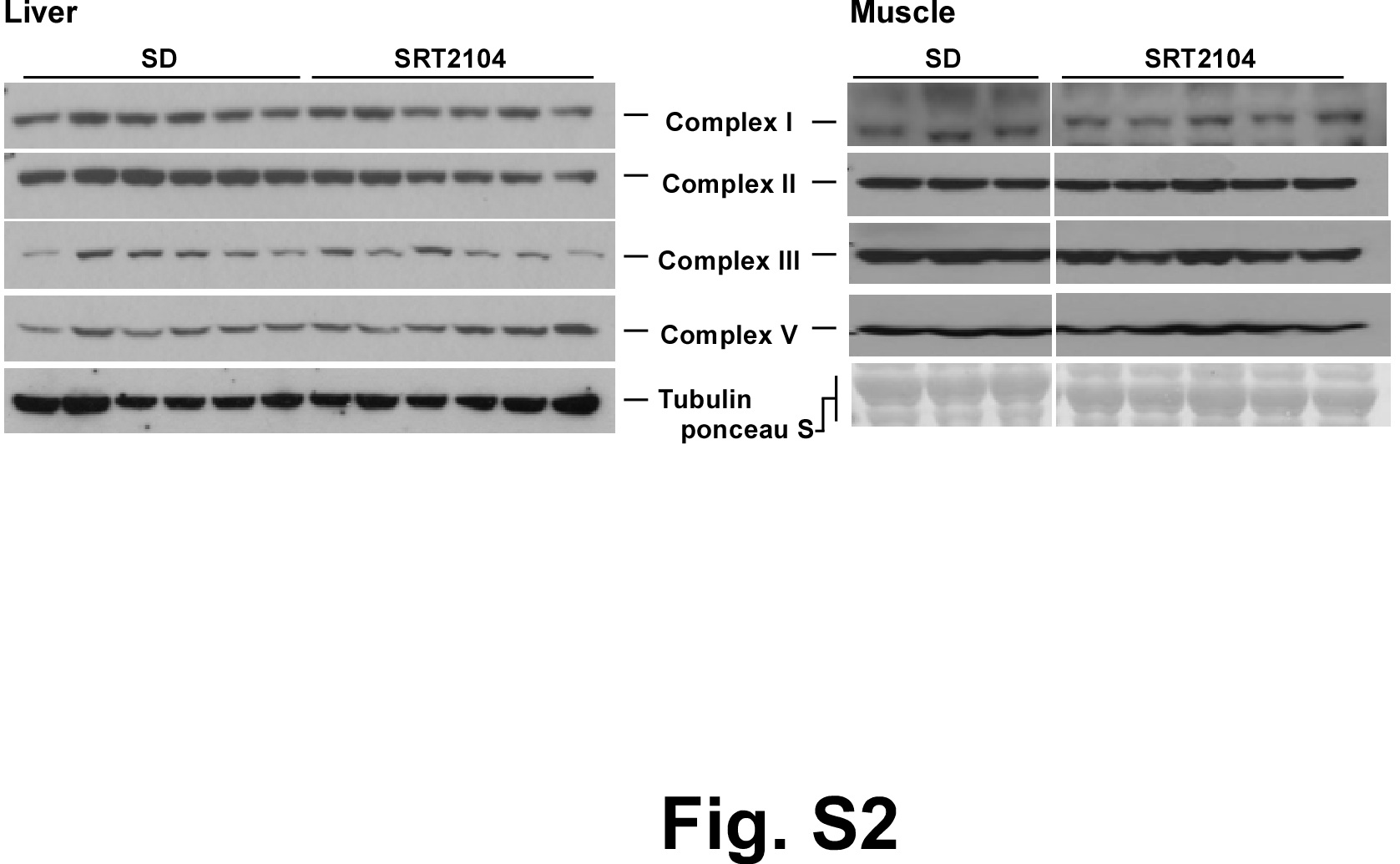

Supplement: Supplementary file 2 — Fig. S2 Representative immunoblots of mitochondrial complexes in liver and muscle from mice on a standard diet (SD) and SD supplemented with SRT2104 (related to Fig. 3). [file acel0013-0787-sd2.jpg]

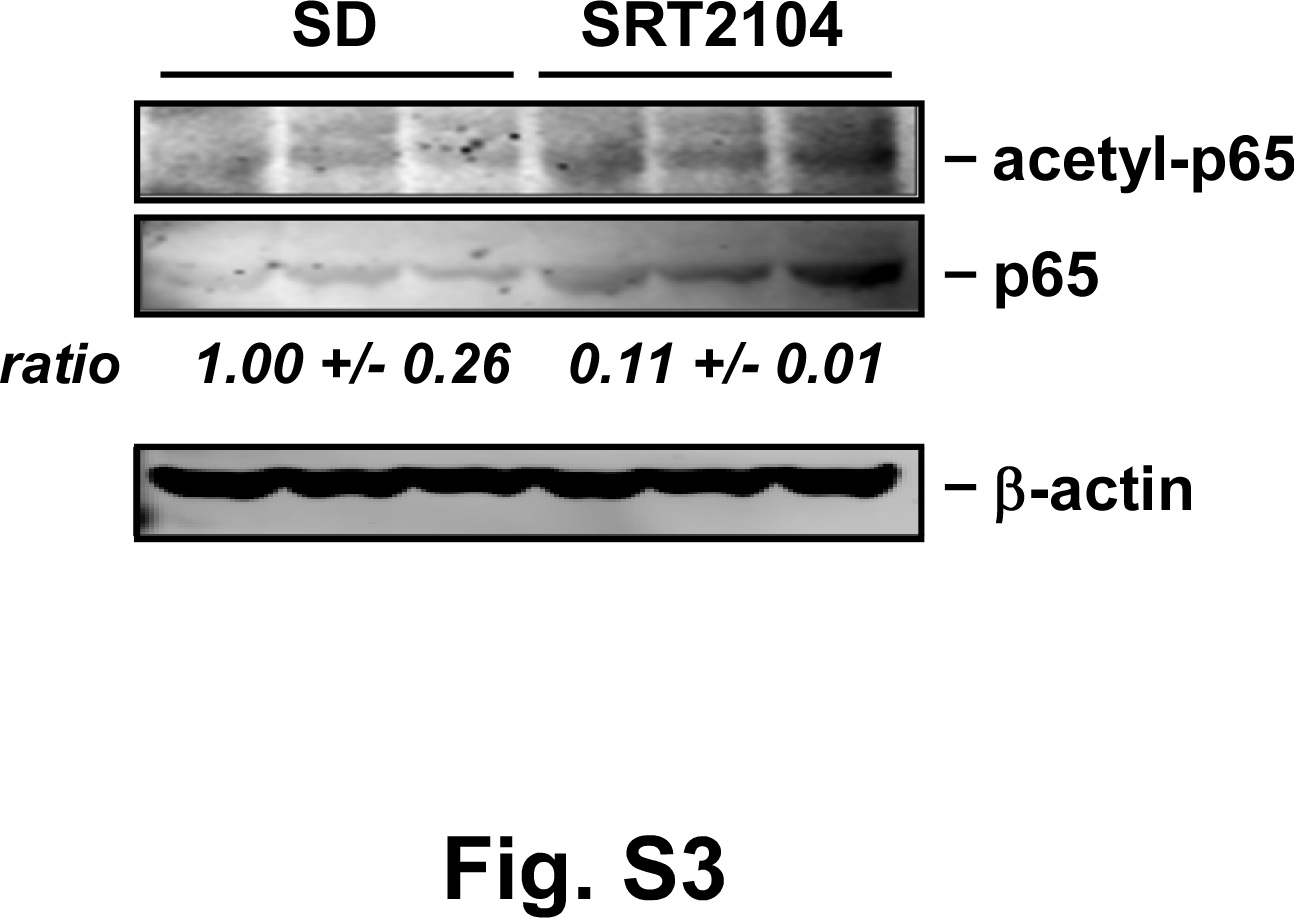

Supplement: Supplementary file 3 — Fig. S3 SRT2104 reduces p65/RelA acetylation levels in C2C12 cells. [file acel0013-0787-sd3.jpg]

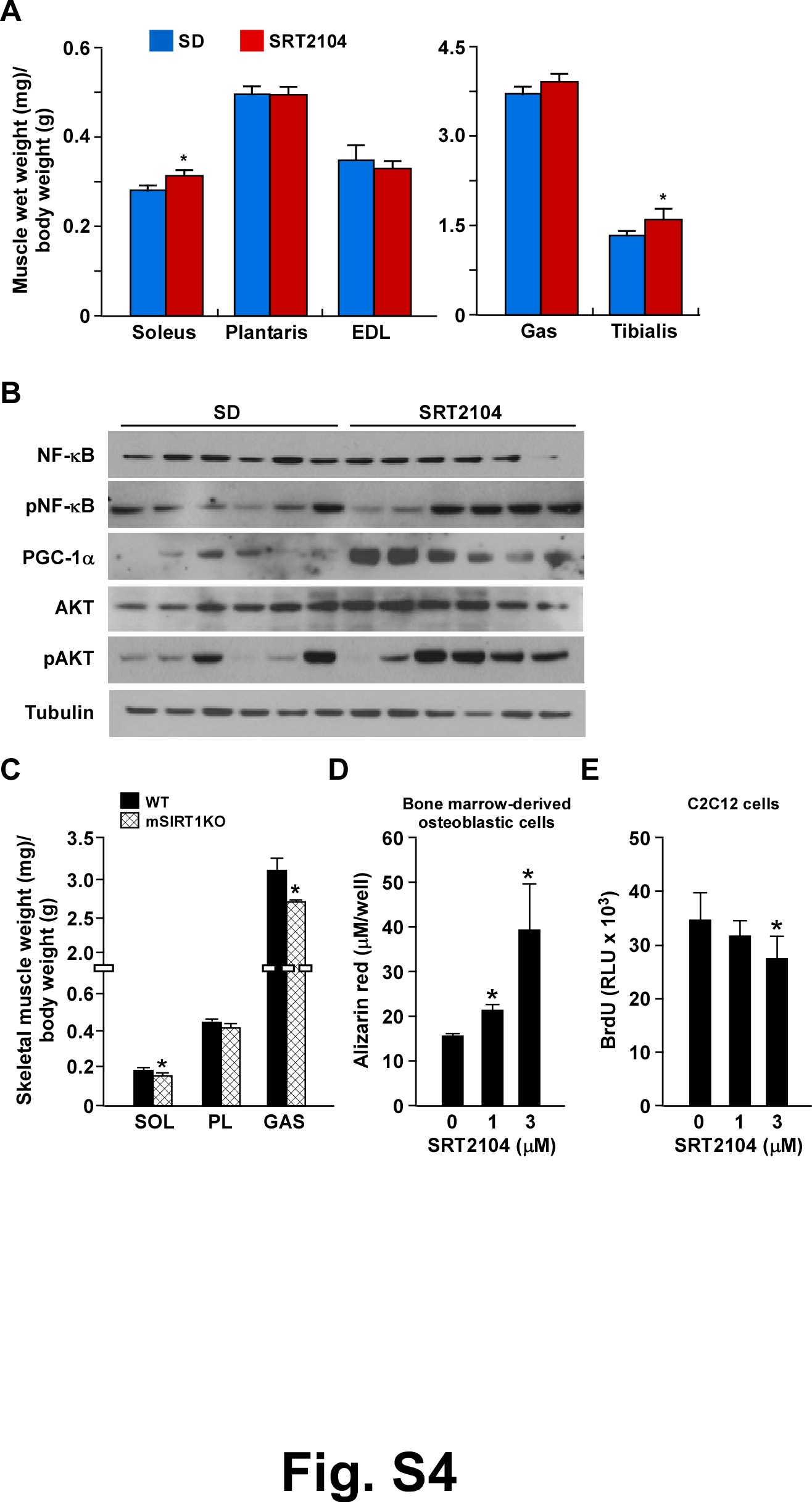

Supplement: Supplementary file 4 — Fig. S4 Impact of SRT2104 supplementation on muscle and bone health (related to Fig. 4). [file acel0013-0787-sd4.jpg]
