# Supplementary material for: SRT2104 extends survival of male mice on a standard diet and preserves bone and muscle mass
Source: Aging Cell. 2014 Jun 16;13(5):787–96. doi: 10.1111/acel.12220 (PMC4172519; doi:10.1111/acel.12220)
Supplement: Supplementary file 5 — Data S1 Material and methods. Table S1 Major gross pathologies identified at necropsy. Table S2 Blinded histopathological analysis. Table S3 Effect of SRT2104 on various biomarkers in serum. Table S4 Body composition. Table S5 The ten most highly upregulated and downregulated genes, based on Z-ratio in liver and muscles of SRT2104-treated mice compared with standard diet (SD). Table S6 List of pathways significantly modified by SRT2104 and CR in the liver of SD-fed mice. Table S7 List of pathways significantly modified by SRT2104 and CR in muscle of SD-fed mice. Table S8 Significant expression of a set of NF-κB target genes in the liver of SRT2104- vs. CR-treated mice. Table S9 Significant expression of a set of NF-κB target genes in skeletal muscle of SRT2104- vs. CR-treated mice. Table S10 List of primer sequences used for quantitative PCR analysis. [file acel0013-0787-sd5.docx]

**Supplementary Materials for**

**SRT2104 extends survival of male mice on a standard diet and preserves bone and muscle mass**

Evi M. Mercken^1†^, Sarah S. Mitchell^1,2,3†^, Alejandro Martin-Montalvo^1^, Robin K. Minor^1^, Maria Almeida^4^, Ana P. Gomes^5^, Morten Scheibye-Knudsen^6^, Hector H. Palacios^1^, Jordan J. Licata^1^, Yongqing Zhang^7^, Kevin G. Becker^7^, Husam Khraiwesh^8^, José A. González-Reyes^8^, José M. Villalba^8^, Joseph A. Baur^9^, George P. Vlasuk^10^, James L. Ellis^10^, David A. Sinclair^5^, Michel Bernier^1^, Rafael de Cabo^1*^.

^1^Translational Gerontology Branch, National Institute on Aging, National Institutes of Health, Baltimore, MD 21224, USA.

^2^Kolling Institute of Medical Research, Royal North Shore Hospital, St Leonards, NSW 2065, Australia.

^3^Sydney Medical School, University of Sydney, Sydney, NSW 2006, Australia.

^4^Division of Endocrinology and Metabolism, Center for Osteoporosis and Metabolic Bone Diseases, University of Arkansas for Medical Sciences and the Central Arkansas Veterans Health Care System, Little Rock, AR 72205, USA.

^5^Glenn Labs for the Biological Mechanisms of Aging, Harvard Medical School, Boston, MA 02115, USA.

^6^Laboratory of Molecular Gerontology, National Institute on Aging, National Institutes of Health, Baltimore, MD 21224, USA.

^7^Gene Expression and Genomics Unit, National Institute on Aging, National Institutes of Health, Baltimore, Maryland 21224, USA.

^8^Departamento de Biología Celular, Fisiología e Inmunología, Universidad de Córdoba, Campus de Excelencia Internacional Agroalimentario ceiA3, Campus Rabanales Edificio Severo Ochoa, 3ª planta, Córdoba, 14014, Spain.

^9^Institute for Diabetes, Obesity, and Metabolism and Department Physiology, Perelman School of Medicine, University of Pennsylvania, PA 19104, USA.

^10^Sirtris, a GSK company, 200 Technology Square, Cambridge, MA 02139, USA.

^†^These authors contributed equally to this research.

^*^Corresponding author: [decabora@grc.nia.nih.gov](mailto:decabora@grc.nia.nih.gov) (R.de C.).

This pdf file includes:

**Material and Methods**

**Supplemental Tables 1 to 10**

**Supplemental Figures 1-4**

**Methods**

**Metabolic assessment.** Mice were housed singly with water and food available *ad libitum* and maintained at ~24°C under a 12:12-hour light-dark cycle (light period 0600-1800). All mice were acclimatized to monitoring cages for 3-6 hours prior to the before recording began. Sample air was passed through an oxygen (O_2_) sensor for determination of O_2_ content. O_2_ consumption was determined by measuring oxygen concentration in air entering the chamber compared with air leaving the chamber. The sensor was calibrated against a standard gas mix containing defined quantities of O_2_, carbon dioxide (CO_2_), and nitrogen (N_2_). Constant airflow (0.6 L/min) was drawn through the chamber and monitored by a mass-sensitive flow meter. The concentrations of O_2_ and CO_2_ were monitored at the inlet and outlet of the sealed chambers to calculate oxygen consumption. Each chamber was measured for 30 seconds at 30-minute intervals and data were recorded for 60 hours total. Movement (both horizontal and vertical) was also monitored. The system has beams 0.5 inches apart on the horizontal plane providing a high-resolution grid covering the XY-planes and the software provides counts of beam breaks by the mouse in 30-second epochs.

**Rotarod.** Mice were given a habituation trial at a constant speed of 4 rpm for 1 minute before the first trial. On the same day there was a total of three trials given, separated by 30-minute rest periods, during which the rotarod accelerated from 4 to 40 rpm over a period of five minutes. Latency to fall was recorded and averaged over all three trials (n=5 SD, n=6 SRT2104; age=80 weeks, diet=40 weeks).

**Treadmill.** Mice were required to exercise on the treadmill until exhaustion. The treadmill was horizontal (0° incline) and mice ran in groups of 6. Subjects were habituated at a constant speed of 4 m/min for 5 minutes on day 1. The following day each mouse was given a trial starting at 7 m/min for 0-3 minutes, 12 m/min for 3-7 minutes, 15 m/min for 7-25 minutes, and 19 m/min for 25 minutes (n=5 SD, n=6 SRT2104; age=80 weeks, diet=40 weeks).

**Serum markers and HOMA calculation.** Plasma concentrations of ALT, AST, CHOL, CRP, LDL and TG were measured using the Cobas Integra 400 automated analyzer (Roche Diagnostics, Indianapolis, IN) (n =4-10 per group; 72 weeks age, 45 weeks diet). Free fatty acids were measured using a kit according to the manufacturers instructions (Roche Diagnostics, Indianapolis, IN) (n=6 SD, n=6 SRT2104; 81 weeks age, 41 weeks diet). Insulin was measured in serum using an enzyme-linked immunosorbent assay (Crystal Chem, Downers Grove, IL) according to the manufacturer’s instructions (n=8 SD, n=6 SRT2104; 72 weeks age, 45 weeks diet). Insulin resistance was calculated from fasted glucose and insulin values using the HOMA2 Calculator software available from the Oxford Centre for Diabetes, Endocrinology and Metabolism, Diabetes Trials Unit website ([www.dtu.ox.ac.uk](http://www.dtu.ox.ac.uk)). Inflammatory markers were measured in a multiplex plate (Millipore, Billerica, MA) according to the manufacturer’s instructions (n=8 SD, n=8 SRT2104; 47 weeks age, 20 weeks diet).

**Electron Microscopy.** Samples were post fixed in 1% osmium tetroxide for 1 hour at 4**°**C in the same buffer, dehydrated and then embedded in Embed 812 resin (Electron Microscopy Sciences, Hatfield PA), after which samples were transferred to pure resin for 24 hours. Blocks were formed in fresh resin contained in silicon molds, and the resin was allowed to polymerize for 48 hours at 65**°**C. Ultrathin sections were obtained and stained with uranyl acetate and lead citrate, and then examined on a Philips CM-10 electron microscope at 25,000 x magnification by a blinded investigator. Ten digital images per sample were analyzed using NIH Image J (NIH, USA) software for measurement of mitochondrial area. Mitochondrial numerical density (Nv) values were obtained following a point analysis according to previously described methods ([Khraiwesh *et al.* 2013](#_ENREF_18)) (n=3 SD, n=3 SRT2104; 81 weeks age, 41 weeks diet).

**PCR.** Detailed information can be found in the supplemental section. For **longevity liver and muscle**, total RNA was extracted from frozen tissue samples using the RNeasy kit (Qiagen). Complementary DNA was synthesized from total RNA with the SuperScript First-Strand Synthesis System (Invitrogen, Carlsbad, CA) and random hexamer primers. The real-time polymerase chain reaction measurement was performed on individual cDNAs by using SYBR green dye to measure duplex DNA formation with the Roche Lightcycler system. The calculation of mRNA expression was performed by the 2^−ΔΔCT^ method normalized to the expression of GAPDH (n=5 SD, n=5 SRT2104; 41 weeks age, 14 weeks diet). For **bone measurements** presented here total RNA was extracted from cultured cells using Ultraspec (Biotecx Laboratories, Houston, TX) and reverse-transcribed using the High-Capacity cDNA Archive Kit (Applied Biosystems, Grand Island, NY), according to the manufacturer’s instructions. Taqman quantitative PCR was performed as previously described ([Almeida *et al.* 2005](#_ENREF_1)) to determine mRNA levels using the following primers: Mm00490758_m1 (SIRT1) and Mm00475528_m1 (ribosomal protein S2) manufactured by the TaqMan^®^ Gene Expression Assays service (Applied Biosystems). mRNA expression levels were normalized to the house-keeping gene ribosomal protein S2 using the Δ∆Ct method ([Livak & Schmittgen 2001](#_ENREF_22)). Primers and probe sequences are listed in Table S10.

**Western blotting.** Tissues were lysed in radioimmunoprecipitation buffer supplemented with ethylenediaminetetraacetic acid and ethylene glycol tetraacetic acid (Boston BioProducts, Ashland, MA) and protease and phosphatase inhibitors (Sigma Aldrich, St Louis, MO). Following centrifugation (14,000 rpm, 30 minutes at 4ºC) protein concentration was quantified using the Bradford assay method (Bio-Rad, Hercules, CA). Proteins were separated by sodium dodecyl sulfate polyacrylamide gel electrophoresis under reducing conditions and then transferred to nitrocellulose membranes. Western blots were performed according to standard methods unless otherwise specified. Membranes were blocked in either 5% bovine serum albumin or 5% milk, and then incubated (overnight at 4ºC or room temperature for 3-4 hours) with the antibody of interest, followed by incubation with a secondary antibody. The visualization of immunoreactive bands was performed using the ECL Plus Western blotting detection system (GE Healthcare, Pascataway, NJ). The quantification was performed by volume densitometry using ImageJ software (National Institutes of Health, Bethesda, MD) and normalization to tubulin, Ponceau S staining (Sigma Aldrich) or GAPDH where specified. Numerous housekeepers were used due to the inherent and uncontrollable variability that can been seen with internal controls such as these. In this study, the primary antibodies were directed against SIRT1 (cat#: S5447-200UL), monoclonal anti-α-tubulin (cat#: T5168-.2ML), (Sigma Aldrich); PGC-1α (cat#: sc-13067), SOD2 (cat#: sc-30080) and GAPDH (cat#: sc-365062), (Santa Cruz Biotechnology, Santa Cruz, CA); pNF-κB Ser 536 (cat#: 3033), Akt (cat#: 9272), pAkt (cat#: 9275), IκBα (cat#: 4812), (Cell Signaling Technology, Beverly, MA); anti-NDUFA9 Complex I (cat#: ab14713), anti-SHDA Complex II (cat#: 14745), anti-UQCRC2 Complex III (cat#: 14745), anti-ATP5A Complex V (cat#: 14748) (Abcam Inc., Cambridge, MA); NF-κβ (cat#: 1546-1) (Epitomics, Burlingame, CA); 4-HNE (cat#: 393206) (EMD-Calbiochem, La Jolla, CA). Secondary antibodies were ECL anti-rabbit IgG HRP (cat#: NC0146699), ECL anti-mouse IgG HRP (cat#: NA931) (GE Healthcare, Pittsburgh, PA) or bovine anti-goat (cat#: sc-2378; Santa Cruz Bioscience, Dallas, TX) where appropriate. For **acetylated NF-κB p65 western blots**, primary antibodies used were NF-κB p65 (L8F6) (cat#: 6956) (Cell Signaling Technology); and anti-NF-κB p65 (acetyl K310) (cat#: ab19870) (Abcam Inc.). Secondary antibodies used Alexa Fluor® 647 donkey anti-rabbit IgG (cat#: A31573), Alexa Fluor® 488 donkey anti-mouse IgG (cat#: A21202) (Invitrogen). Bands were visualized using the Typhoon Variable Mode Imager (Amersham Biosciences, Sunnyvale, CA). The quantification was performed by volume densitometry using ImageJ software and normalization to actin (cat#: 4970; Cell Signaling Technology). For **bone measurements** presented in this study, SIRT1 and β-actin levels in cultured osteoclasts were determined using a rabbit polyclonal antibody for SIRT1 (cat#: sc-15404) and a mouse monoclonal antibody for β-actin (cat#: sc-47778) (Santa Cruz Biotechnology, Dallas, TX). ([Rocha-Martins *et al.* 2012](#_ENREF_38)). Antibodies were used generally at a dilution recommended by the manufacturer. **Protein carbonylation** was measured using an oxyblot protein oxidation kit (Millipore, Billerica, MA) according to the manufacturer’s instructions (**Longevity study:** n=3-6 per group; 41 weeks age, 14 weeks diet, n=6 SD, n=6 SRT2104; 81 weeks age, 41 weeks diet; **Hindlimb suspension:** n=6 SD, n=6 SRT2104; 26 weeks age, 6 weeks diet).

**Bone imaging.** Micro-CT analysis was performed on femoral bones from mice as indicated. Briefly, four and eight days prior to sacrifice, mice received an i.p. injection of tetracycline (33 mg/kg, Sigma Aldrich, St-Louis, MO). Femurs were loaded into 10 mm diameter scanning tubes and imaged with a Scanco microCT40 instrument (CT40, Scanco Biomedical, Bruttisellen, Switzerland) as previously described ([Jilka *et al.* 2010](#_ENREF_16); [Martin-Millan *et al.* 2010](#_ENREF_23)). Cortical and trabecular bone measurements were analyzed as previously described ([Martin-Millan *et al.* 2010](#_ENREF_23); [Onal *et al.* 2012](#_ENREF_31)) (**Longevity study:** n=5 SD, n=5 SRT2104; 81 weeks age, 41 weeks diet; **Hindlimb suspension study:** n=5 SD, n=5 SRT2104; 26 weeks age, 6 weeks diet; **Whole body SIRT1 KO:** n=9 WT, n=10 KO; 32 weeks age).

**Mineralization assay.** Freshly isolated murine bone marrow cells pooled from three mice were seeded on 12-well tissue culture plates at 5 × 10^6^ cells per well in standard culture medium and cultured for 10 days. One half of the medium was replaced every 5 days. FBS was then reduced to 2% and SRT2104 was added to the cultures. Two days later, 10 mM β-glycerophosphate was added to the medium. The mineralization matrix was stained with 40 mM alizarin red solution two weeks later.

**Proliferation assay.** C2C12 cells seeded in a 96-well plate were incubated with vehicle or SRT2104 for 72 hours. BrdU was added at a final concentration of 10 µM for 24 hours, and BrdU incorporation was then determined by ELISA chemiluminescence (Roche Diagnostics, Indianapolis, IN).

**SUPPLEMENTARY TABLES**

**Table S1.** Major gross pathologies identified at necropsy.

|  |  | **Diet Treatment (n)** | |
| --- | --- | --- | --- |
|  |  | **SD (87)** | **SRT2104 (89)** |
| Mean age of death (weeks) | | 113 ± 3 | 124 ± 2 |
| Heart | Enlarged | 9 | 3^#^ |
|  | Pericardial fat | 2 | 1 |
|  | Ischemic foci | 7 | 5 |
| Kidney | Peri-renal fat | 9 | 1* |
| Liver | Hepatocarcinoma | 9 | 3^#^ |
|  | Steatosis | 2 | 1 |

The percentage of mice presenting with various pathologies is represented. SD, standard diet.

^#^ p = 0.078 compared to SD; * p < 0.05 compared to SD. Analysed using Fisher’s Exact Test.

**Table S2.**  Blinded histopathological analysis.

|  |  | **SD** | **SRT2104** |
| --- | --- | --- | --- |
| **Liver** | Lymphocyte infiltration | 1 ± 0 (67) | 1 ± 0 (17) |
|  | Fatty change | 2.3 ± 0.3 (67) | 2.0 ± 0 (33) |
| **Kidney** | Lymphocyte infiltration | 1.6 ± 0.3 (83) | 1.8 ± 0.3 (100) |
|  | Glomerulonephritis | 2.0 ± 0.4 (50) | 1.7 ± 0.3 (100) |
| **Heart** | Lesion | 0 | 0 |
| **Spleen** | Congestion | 6 (100) | 6 (100) |

Lymphocyte infiltration and fatty change are scored on a scale of 1-4 (least to most severe); data is mean ± SEM (%). Heart and spleen data is represented as number of cases occurring. 81 weeks age, 41 weeks diet, n=6 per group. SD, standard diet.

**Table S3.** Effect of SRT2104 on various biomarkers in serum.

|  | **Diet Treatment** | |
| --- | --- | --- |
|  | **SD** | **SRT2104** |
| ALT (U/L) | 90 ± 26 | 80 ± 43 |
| AST (U/L) | 270 ± 35 | 86 ± 9^*^ |
| CHOL (mg/dL) | 221 ± 12 | 196 ± 6 |
| CRP (mg/dL) | 0.04 ± 0.03 | 0.03 ± 0.01 |
| LDL (mg/dL) | 40 ± 5 | 34 ± 3 |
| TG (mg/dL)  FFA (mM) | 106 ± 14  0.55 ± 0.03 | 120 ± 9  0.41 ± 0.07 |

Data is presented as mean ± SEM; ^*^p < 0.05 compared to standard diet (SD). ALT, alanine aminotransferase; AST, aspartate aminotransferase; CHOL, total cholesterol; CRP, C-reactive protein; LDL, low density lipoprotein; TG, triglycerides (n=4-10 per group, 72 weeks age, 45 weeks diet); FFA, free fatty acids (n = 6 SD, n = 6 SRT2104, 81 weeks age, 41 weeks diet).

**Table S4.** Body composition.

|  | **Diet treatment** | |
| --- | --- | --- |
|  | **SD** | **SRT2104** |
| Body weight (g) | 41.3 ± 1.5 | 38.3 ± 0.7 |
| Fat mass (g) | 9.8 ± 0.7 | 7.9 ± 0.4^*^ |
| Lean mass (g) | 23.5 ± 0.8 | 22.0 ± 0.5 |
| Fat (%) | 23.5 ± 0.9 | 20.4 ± 0.8^*^ |
| Lean (%) | 57.0 ± 0.4 | 57.5 ± 0.4 |
| Lean:fat ratio | 2.5 ± 0.1 | 2.4 ± 0.0 |

Data is presented as mean ± SEM; ^*^p < 0.05 compared to standard diet (SD) (n=15 SD, n=13 SRT2104, 76 weeks age; 49 weeks diet).

**Table S5.** The ten most highly up-regulated and down-regulated genes, based on Z-ratio in liver and muscles of SRT2104 treated mice compared to standard diet (SD). The effect of calorie restriction (CR) on the same genes is also shown.

| **Gene** | **Z-ratio, liver** | **Z-ratio, liver** | **Gene** | **Z-ratio, muscle** | **Z-ratio, muscle** |
| --- | --- | --- | --- | --- | --- |
| **Symbol** | **SRT2104:SD** | **CR:SD** | **Symbol** | **SRT2104:SD** | **CR:SD** |
| Rgs16 | **14.05** | **5.73** | Cish | **7.55** | **11.58** |
| Egr1 | **12.02** | **7.1** | Chac1 | **5.59** | *0.47* |
| Dbp | **11.38** | **-4.97** | Dbp | **4.64** | *-0.3* |
| Ctgf | **8.96** | **3.27** | Atp1a2 | **4.38** | **3.22** |
| Cyp1a2 | **8.08** | *0.37* | Preb | **4.37** | *0.5* |
| Usp2 | **8.02** | **6.93** | Aup1 | **4.25** | *-0.53* |
| Socs2 | **7.79** | *4.78* | Hnrpl | **4.22** | **4.67** |
| Hhex | **7.77** | **6.0** | Ccdc85b | **4.13** | *-0.15* |
| Txnip | **7.63** | **5.18** | 260000IBIRik | **4.09** | **-2.25** |
| Chrna4 | **7.48** | *-1.35* | Gart | **4.08** | **1.6** |
| Bhmt | **-9.8** | *-1.65* | Hspa8 | **-13.24** | **-5.4** |
| Clpx | **-8.26** | **-2.24** | Pdlim5 | **-11.82** | *-0.05* |
| Clpx | **-7.22** | **-4.49** | Loc100046918 | **-10.89** | *0.02* |
| Insig2 | **-7.1** | **-10.43** | Loc669660 | **-10.74** | *0.66* |
| Lrfn3 | **-7.09** | **-2.70** | Cfd | **-9.58** | *8.94* |
| C730036D15Rik | **-6.79** | *0.37* | Itm2a | **-9.16** | *0.34* |
| Hspb1 | **-6.44** | *2.3* | Tspan3 | **-8.95** | **-2.31** |
| Rsad2 | **-6.15** | **-5.09** | Ppp1ca | **-8.87** | **-1.9** |
| Elovl3 | **-6.11** | **-9.58** | Casq1 | **-8.77** | *-0.34* |
| Gadd45a | **-6.04** | **4.53** | Arl6ip5 | **-8.73** | *0.50* |

All Z-ratio values statistically significant are in **Boldface**.

**Table S6.** List of pathways significantly modified by SRT2104 and CR in the liver of SD-fed mice.

**Please see supplemental file.**

**Table S7.** List of pathways significantly modified by SRT2104 and CR in muscle of SD-fed mice.

**Please see supplemental file.**

**Table S8.** Significant expression of a set of NF-κB target genes in the liver of SRT2104- vs. CR-treated mice

**Please see supplemental file.**

**Table S9.** Significant expression of a set of NF-κB target genes in skeletal muscle of SRT2104- vs. CR-treated mice

**Please see supplemental file.**

**Table S10.** List of primer sequences used for quantitative PCR analysis.

| **Gene** | **Forward primer sequence** | **Reverse primer sequence** |
| --- | --- | --- |
| COMMD1 | AAAAAGCAAGGTGGCATCAC | GTTGAGTGCCGTGACTGAGA |
| COMMD3 | AAAGCATTGTCATGCAGCAG | AAGCGCCAAGAAACATCAGT |
| COMMD10 | GCTGGACTCCCTCACATGAT | GAGAAGGCACACTCGAAACC |
| GAPDH | CACCAACTGCTTAGCCCC | TCTTCTGGGTGGCAGTGATG |
| β-actin | ACCTTCTACAATGAGCTGCG | CTGGATGGCTACGTACATGG |
