# Supplementary material for: SRT2104 extends survival of male mice on a standard diet and preserves bone and muscle mass
Source: Aging Cell. 2014 Jun 16;13(5):787–96. doi: 10.1111/acel.12220 (PMC4172519; doi:10.1111/acel.12220)
Supplement: Supplementary file 10 [file acel0013-0787-sd10.docx]

**Supplemental Figure Legends**

**Figure S1.** Effects of SRT2104 supplementation on various metabolic parameters in mice on a standard diet (Related to Figure 1). **(A)** Food consumption measured bi-weekly over the course of the study for mice fed a standard diet (SD) or SD supplemented with SRT2104; **(B)** Oxygen consumption (VO_2_); **(C)** Respiratory exchange ratio (RER); **(D)** Oral glucose tolerance test; and **(E)** Insulin tolerance test. Data are mean ± SEM. ^*^p<0.05.

**Figure S2.** Representative immunoblots of mitochondrial complexes in liver and muscle from mice on a standard diet (SD) and SD supplemented with SRT2104 (Related to Figure 3).

**Figure S3.** SRT2104 reduces p65/RelA acetylation levels in C2C12 cells. Cells were treated with vehicle (0.1% DMSO) or 3 µM SRT2104 for 24 h. Representative immunoblots for p65, Ac-p65 and actin are shown. The ratio of acetylated/total form of p65/RelA is shown. Data are mean ± SEM (p=0.025) (Related to Figure 3).

**Figure S4.** Impact of SRT2104 supplementation on muscle and bone health (Related to Figure 4). **(A)** Muscle weight following a 48-h fast in mice fed either a standard diet (SD) or SD supplemented with SRT2104 for 6 weeks; **(B)** Representative immunoblots in gastrocnemius muscles; **(C)** Muscle weights after 14 d of hindlimb suspension of 6-months-old wild-type (WT) mice and muscle-specific SIRT1-KO mice (mSIRT1KO); (**D**) Proliferation rate in C2C12 myoblasts after SRT2104 treatment; and (**E**) Mineralization in bone marrow-derived osteoblastic cells after SRT2104 treatment (1 and 3 µM) for 15 d. Data are mean ± SEM. ^*^p<0.05.
